# STAT3 c.1915C > T variant-associated Hyper-IgE syndrome in a child: a case report

**DOI:** 10.3389/fped.2025.1693297

**Published:** 2025-12-12

**Authors:** Qianqian Dai, Yanan Wang, Zhiyuan Wang, Liang Ru

**Affiliations:** Department of Pediatrics, The First Affiliated Hospital of Xinjiang Medical University, Urumqi, Xinjiang, China

**Keywords:** case report, Hyper-IgE syndrome, IgE, primary immunodeficiency, STAT3 gene mutation

## Abstract

**Background:**

Hyper-IgE syndrome (HIES) is a rare primary immunodeficiency disorder characterized by severe eczema, recurrent skin and respiratory infections, and markedly elevated serum IgE levels. Early diagnosis and management are crucial for prognosis, but clinical misdiagnosis is common due to its rarity. We report a case of a 6-year-old girl with HIES, where a rarely reported STAT3 mutation was discovered through genetic testing, aiming to enhance disease awareness and diagnostic accuracy.

**Methods:**

The patient underwent comprehensive history taking, physical examination, serum IgE measurement, and ancillary tests. Whole-exome sequencing via next-generation sequencing (NGS) was used to analyze the STAT3 gene, with mutation validation by Sanger sequencing. Novelty was assessed against the Genome Aggregation Database (gnomAD). Management included anti-infective therapy, skin care, nutritional support, and a long-term home care plan.

**Results:**

The child presented with a 5-year history of eczema and skin pustules, growth delay, and malnutrition, along with significantly elevated serum IgE. Genetic testing revealed a heterozygous c.1915C > T missense mutation in STAT3, which was unreported in gnomAD and consistent with HIES pathogenicity based on literature. Supportive care, including anti-infectives and skin management, led to substantial improvement, and long-term home care was implemented post-discharge.

**Conclusion:**

The STAT3 c.1915C > T mutation is likely causative of HIES, expanding its genetic mutation spectrum. This case highlights the critical role of genetic testing in rare disease diagnosis and the importance of integrating acute treatment with long-term home care, providing insights for improved early detection and management of HIES.

## Introduction

The term “Hyper-IgE Syndrome” (HIES) was formally proposed in 1974 when Hill et al. recognized that Job syndrome (reported in 1966) and Buckley syndrome (1972) represented the same disease entity, unifying them under the nomenclature “HIES” (1). HIES is a rare primary immunodeficiency characterized by elevated serum IgE, recurrent pneumonia with pneumatoceles, and recurrent staphylococcal skin abscesses (2). It affects diverse ethnic groups, typically presenting in childhood with heterogeneous clinical manifestations that severely impact physical and psychological health (3, 4). Due to its rarity, many clinicians lack familiarity with HIES, leading to frequent misdiagnosis as common conditions like atopic dermatitis or allergic disorders (5).

HIES follows autosomal dominant (AD) or autosomal recessive (AR) inheritance patterns, with STAT3-mediated dominant-negative (DN) mutations being the most common genetic etiology for AD-HIES (6). Early diagnosis, infection management, and supportive care are critical for prognosis. Next-generation sequencing (NGS) has facilitated identification of novel mutations and refined diagnostic approaches (7). Integrating clinical features with genetic testing enables accurate early diagnosis, guiding tailored treatments to improve patients' quality of life (8).

We describe a 6-year-old girl of Uyghur ethnicity from Xinjiang, China, diagnosed with HIES via NGS. A heterozygous STAT3 c.1915C > T missense mutation—unreported in clinical case literature—was identified, highlighting HIES as a key differential diagnosis in patients with recurrent skin infections, eczema, and related symptoms.

## Case presentation

**Figure 1:**
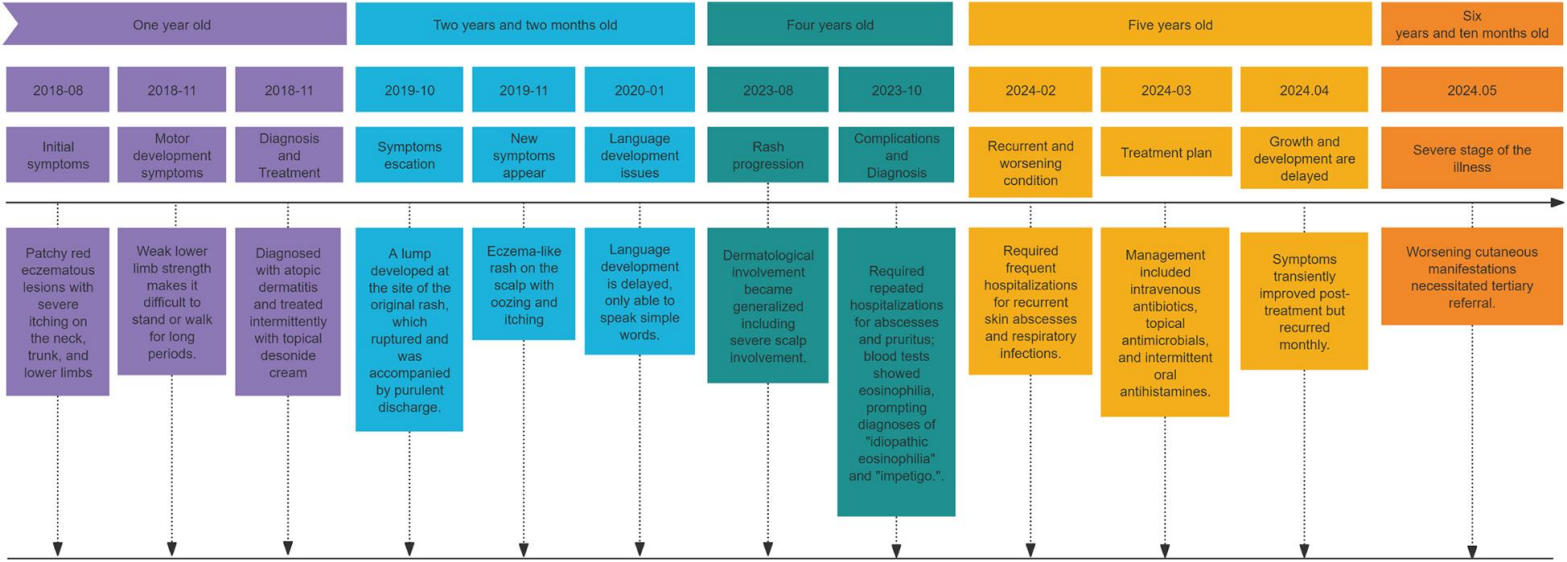
Timeline of disease progression and key clinical events. This timeline summarizes the major clinical milestones of the patient from symptom onset to diagnosis. Key events include: the development of eczematous patches and lower limb weakness at age 1; progression to fluctuant subcutaneous nodules and scalp involvement at age 2 years 2 months; generalization of dermatological lesions and repeated hospitalizations with eosinophilia at age 4; frequent hospitalizations for recurrent skin and respiratory infections at age 5; and eventual tertiary referral due to worsening manifestations at age 6 years 10 months.

The case presentation, as shown in Figure 1, a 6-year-10-month-old girl presented to our medical center with a 5-year history of recurrent generalized eczema and multiple skin pustules.

Age 1: Developed irregular, scaly erythematous patches (largest 5  ×  7 cm) on neck, trunk, and lower extremities with intense pruritus. Diagnosed with atopic dermatitis and treated intermittently with topical desonide cream. Concurrently exhibited lower limb weakness preventing prolonged standing/walking. Age 2 years 2 months: Progressed to fluctuant subcutaneous nodules (largest 4 × 5 cm) that spontaneously ruptured with purulent discharge. Eczematous lesions with exudate appeared on the scalp. Age 4: Dermatological involvement became generalized including severe scalp involvement. Required repeated hospitalizations for abscesses and pruritus; blood tests showed eosinophilia, prompting diagnoses of “idiopathic eosinophilia” and “impetigo.” Age 5: Required frequent hospitalizations for recurrent skin abscesses and respiratory infections. Management included intravenous antibiotics, topical antimicrobials, and intermittent oral antihistamines. Symptoms transiently improved post-treatment but recurred monthly. Age 6 years 10 months: Worsening cutaneous manifestations necessitated tertiary referral.

Ancillary History: Third child of non-consanguineous healthy parents. Birth weight: 2,500 g (full-term spontaneous vaginal delivery). No family history of immunodeficiency or HIES-like disorders. Normal skin and development during infancy.

## Results

### Physical examination

**Figure 2:**
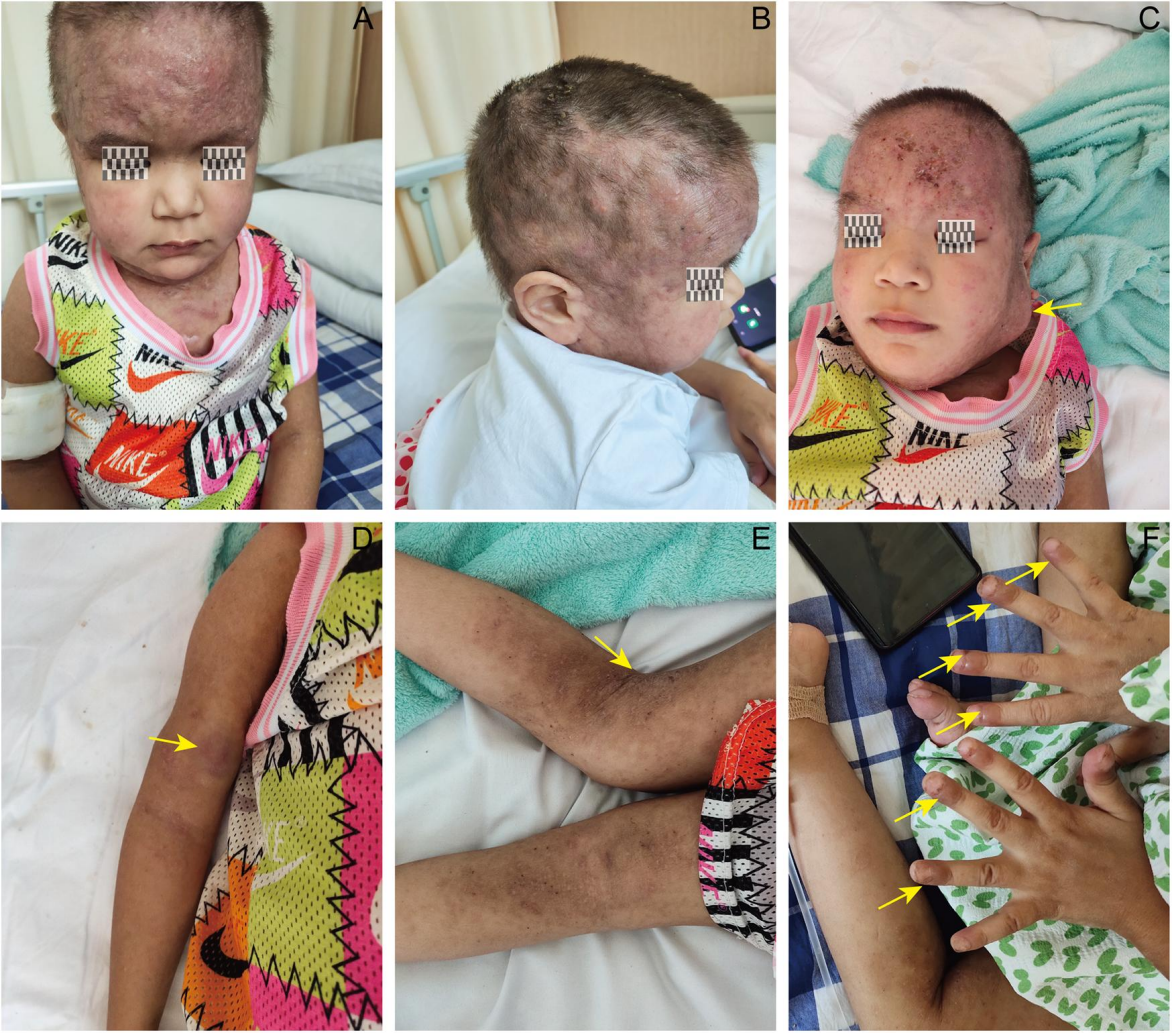
Clinical and dysmorphic features of the patient with STAT3-HIES. Facial photograph demonstrating characteristic craniofacial dysmorphism, including a prominent forehead, depressed nasal bridge, and broad nasal base. **(B)** Extensive eczematous dermatitis and erythematous plaques on the scalp and forehead. **(C)** Arrow indicates a 5 × 5 cm “cold” abscess in the left mandibular region. **(D)** Arrow indicates a 3 × 2 cm “cold” abscess on the right upper arm. **(E)** Lower limb muscular atrophy and knee hyperextension. **(F)** Arrows point to multiple “cold” abscesses at the distal interphalangeal joints.

Persistent developmental delays included: Severe growth retardation (height 100 cm, weight 13 kg). Motor impairment: Inability to sit/walk independently (Grade 4 muscle strength in lower limbs; muscle atrophy noted). Speech delay: Limited to single words (5-year history). Craniofacial dysmorphism: Prominent forehead, hypertelorism, depressed nasal bridge, broad nasal base. Dermatological: Scalp and forehead with extensive erythematous, pruritic plaques; fluctuant nodules (left maxillofacial: 5 × 5 cm; right arm: 3 × 2 cm) without local warmth or tenderness. Lymphadenopathy: Non-tender, mobile lymph nodes in cervical, axillary, and inguinal regions. Musculoskeletal: Knee hyperextension, lower limb muscular atrophy, decreased muscle tone. Neurological: Expressive aphasia (inability to form sentences). NIH-HIES score: 73. The patient characteristics are illustrated in Figure 2.

### Laboratory investigations

**Figure 3:**
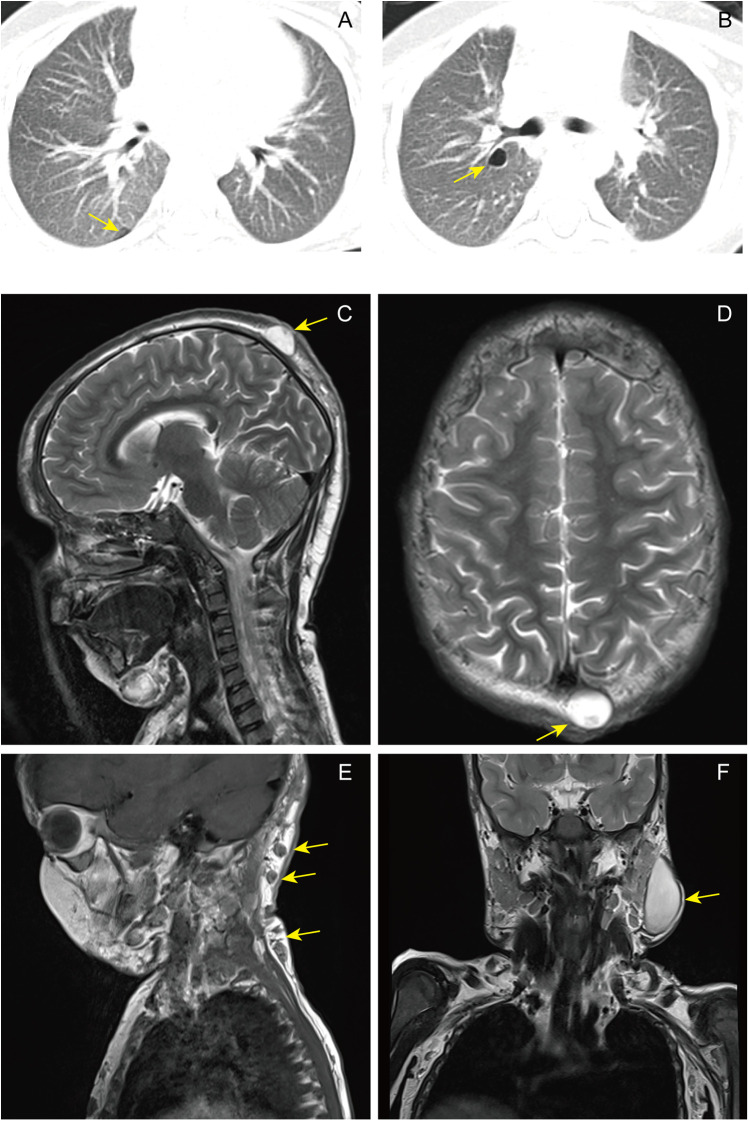
Radiological findings of pulmonary and subcutaneous manifestations in this case. **(A,B)** Axial chest CT images (lung window) demonstrate characteristic pulmonary pneumatoceles. **(A)** A localized hyperlucent area is visible in the right lower lobe posterior basal segment. **(B)** A round, thin-walled, well-defined air cyst with an absence of lung markings is present in the right lower lobe dorsal segment. **(C–F)** Magnetic resonance imaging (MRI) reveals subcutaneous “cold” abscesses in the head and neck region. **(C)** Sagittal T2-weighted image shows a well-circumscribed, round hyperintense lesion in the left parietal scalp. **(D)** Axial T2-weighted image confirms the round, hyperintense lesion in the left parietal subcutaneous tissue. **(E)** Sagittal T1-weighted image demonstrates multiple round, isointense lesions in the occipitocervical subcutaneous tissue. **(F)** Coronal T2-weighted image shows an oval, hyperintense (long T2) lesion in the left lower jaw area subcutaneous tissue.

Laboratory tests of this pediatric patient revealed elevated white blood cell count and increased neutrophil percentage. The eosinophil count was notably high at 18%. The serum IgE level reached an astonishing 22,800 IU/mL. Chest CT imaging demonstrated pulmonary air cysts and linear opacities in the upper lobe of the right lung, as illustrated in Figures 3A,B. MRI of the head, occipitocervical and left lower jaw area subcutaneous tissue revealed abscess formation, as shown in Figures 3C–F. The key laboratory and imaging findings are summarized in Supplementary Table S1. The patient underwent surgical debridement and drainage of abscesses located in the left lower jaw and the right upper arm. Microbiological culture of the drained fluid confirmed methicillin-sensitive Staphylococcus aureus infection. Histopathological examination of the tissue from the right upper arm revealed hyperplastic fibrous and adipose tissue, granulation tissue formation, acute and chronic inflammatory cell infiltration, microabscesses, scattered eosinophilic infiltration, and histiocytic proliferation, as depicted in Supplementary Figures S1A,C. Additionally, bone marrow aspiration and biopsy were performed at the posterior superior iliac spine. Both procedures showed a marked increase in eosinophil count. Bone marrow histopathological findings are presented in Supplementary Figures S1B,D.

### Genetic diagnostic report

**Figure 4:**
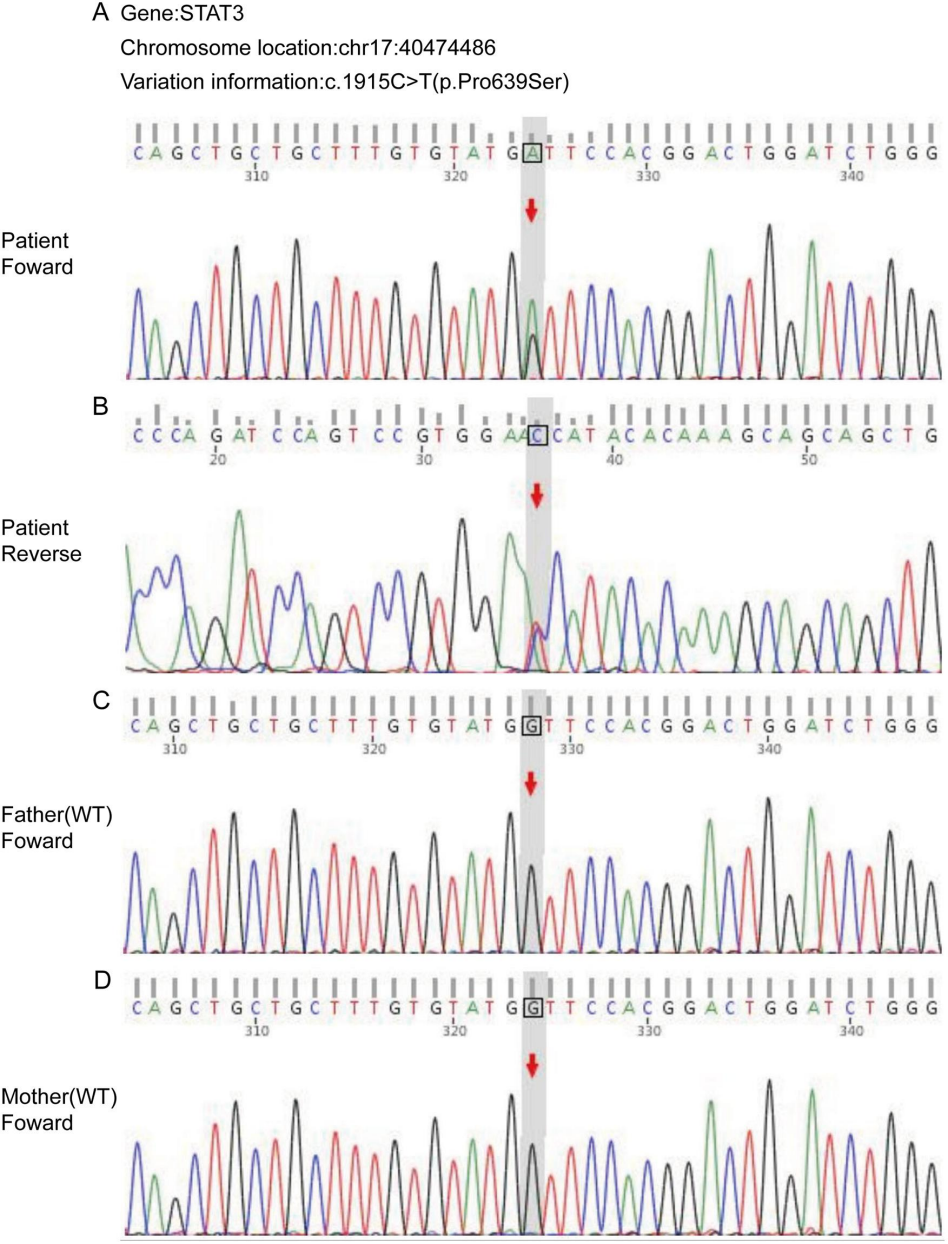
Sanger sequencing validation of the *de novo* STAT3 missense variant c.1915C > T (p.Pro639Ser). Sequencing chromatograms confirm the presence of a heterozygous missense mutation in the proband and its *de novo* origin. The mutation is described as NM_139276.3:c.1915C > T on the cDNA level, indicating a cytosine to thymine substitution at nucleotide position 1915. This results in the amino acid change p.Pro639Ser at the protein level, where proline is replaced by serine at residue 639 within the critical SH2 domain. **(A,B)** Forward and reverse sequence traces from the proband, respectively. The arrow in panel B highlights the heterozygous C-to-T substitution in the reverse sequence. **(C,D)** Sequence traces from the father **(C)** and mother **(D)** at the identical locus, both exhibiting only the wild-type (WT) C nucleotide, thereby confirming the *de novo* inheritance of the variant in the proband.

Considering the rarity of the child's disease, we conducted next-generation sequencing. The results indicated a heterozygous NM_139276.3:c.1915C > T (p.Pro639Ser) missense variant in the coding region of the STAT3 gene. Sanger sequencing confirmed that this *de novo* variant was absent in the peripheral blood samples of the parents. The detailed genetic analysis confirming the heterozygous STAT3 variant is presented in Figure 4. This variant was not listed in the Genome Aggregation Database (gnomAD), supporting its rarity.

According to the American College of Medical Genetics and Genomics (ACMG) guidelines, this variant is classified as “Likely Pathogenic” (9). The supporting evidence includes: its *de novo* status (PS2), location within a well-established mutational hot spot and critical functional domain (SH2 domain) (PM1), absence in population databases (PM2), and concordant deleterious predictions from multiple computational tools (PP3). The patient's phenotype is highly specific for STAT3-HIES (PP4), further corroborating its pathogenic role. Based on the comprehensive medical history, auxiliary examinations, and the conclusive genetic finding of a *de novo*, likely pathogenic STAT3 variant, the final diagnosis was “Hyper-IgE Syndrome, cold abscess of the skin, anemia, and methicillin-sensitive Staphylococcus aureus infection”.

### Diagnosis and differential diagnosis

The patient was ultimately diagnosed with AD-HIES caused by a *de novo* heterozygous STAT3 mutation (c.1915C > T, p.Pro639Ser). The diagnosis was based on a characteristic NIH-HIES score (73), markedly elevated serum IgE (22,800 IU/mL), eosinophilia, recurrent cold abscesses and eczema, distinctive facies, pulmonary cysts, skeletal abnormalities, and confirmation by genetic sequencing.

Prior to confirmation, a systematic approach was taken to rule out key differential diagnoses:

Parasitic Infections: Given the marked eosinophilia and eczema, a comprehensive parasitic antibody panel (including Cysticercus, Paragonimus, and Echinococcus) was performed, with all results returning negative, effectively excluding parasitic infections as a cause of secondary IgE elevation.

Other Genetic Forms of HIES/Combined Immunodeficiency (CID): Although conditions like DOCK8 deficiency (now classified as CID) can present with high IgE and eczema, our patient's history was dominated by bacterial and fungal infections without severe viral infections, aligning more with STAT3-HIES. Furthermore, next-generation sequencing did not identify mutations in other known HIES-associated genes such as ZNF341 or IL6ST.

Severe Atopic Dermatitis and Hematologic Malignancies: While the patient had severe eczema, the co-occurrence of pathologic fractures, pulmonary cysts, and characteristic facies pointed beyond classic atopic dermatitis. Bone marrow aspiration and imaging studies revealed no evidence to support hematologic malignancies like lymphoma.

### Therapeutic intervention

The treatment plan includes intravenous administration of linezolid, methylprednisolone sodium succinate, cimetidine, calcium gluconate, and oral administration of levocetirizine hydrochloride. Surgical debridement and drainage were performed for the abscess in the left lower jaw and the abscess in the right upper arm. In terms of skin care, we disinfected the ruptured area of the abscess and applied mupirocin ointment externally for anti-infection treatment. For the rash on the ulcerated area, we applied Clidorol ointment (twice a day) and 2% chlorhexidine gluconate skin disinfectant (three times a day) to the child. After treatment, the child's skin infection was brought under control, the skin at the site of abscess debridement healed well, and the rash all over the body significantly subsided. After the condition stabilized, the discharge procedures were completed. Outside the hospital, we provided the child with long-term oral trimethopridine-sulfamethoxazole (20 mg/kg, p.o., bid) for infection prevention treatment, and gave oral levcetirizine hydrochloride for up to 2 months. We also persisted in following up and guiding the child's family members to help with skin care. Discharge follow-up: After discharge, the child still experienced intermittent recurrent rashes and cold abscesses on the skin, but the frequency of hospitalization due to severe skin or lung infections decreased to one-third of the previous level.

### Long-term management and follow-up outcomes

We also persisted in following up and guiding the child's family members to help with skin care. Clinical monitoring included regular assessments of skin involvement, growth parameters, and annual chest CT. Excellent adherence to prophylactic medication was reported by the family, with no notable adverse effects. Over the follow-up period, the patient showed marked improvement in general mental state, appetite, and activity tolerance. Consequently, the frequency of hospitalizations for severe skin or pulmonary infections reduced to one-third of the pre-diagnosis level, and family confidence in managing the condition was significantly enhanced.

### Patient and family perspective

The family shared: “After years of navigating multiple hospitals, the recurrent rashes and infections left us feeling helpless. The genetic test finally gave us answers. Knowing it's a lifelong condition is challenging, but having a clear management plan has brought us peace and hope.”

## Discussion

HIES represents a rare and complex primary immunodeficiency disorder characterized by a triad of markedly elevated serum IgE levels, recurrent staphylococcal skin abscesses, and recurrent pneumonia leading to pneumatocele formation. Among the various genetic etiologies identified, autosomal dominant mutations in the STAT3 gene (STAT3-HIES or AD-HIES) constitute the most common and well-characterized subtype. The diagnosis often relies on a combination of clinical features, immunological findings, and genetic confirmation. Woellner et al. established a diagnostic scoring system where a score exceeding 30 points, coupled with specific clinical manifestations such as neonatal eczema, characteristic facies, pathological fractures, and elevated IgE, raises strong suspicion for HIES (10). A score greater than 40 points is considered highly indicative, and the presence of a dominant-negative STAT3 mutation confirms the diagnosis (11). Our patient presented with a compelling clinical picture, including an extraordinarily high NIH-HIES score of 73 points, significantly elevated serum IgE (22,800 IU/mL), recurrent “cold” skin abscesses, pneumatoceles, severe eczematous dermatitis, and characteristic facial features, thereby strongly suggesting STAT3-HIES. Definitive confirmation was obtained through genetic testing, which identified a heterozygous c.1915C > T (p.Pro639Ser) missense variant in the STAT3 gene.

The STAT3 protein is a crucial transcription factor belonging to the STAT family, playing a pivotal role in the signal transduction of numerous cytokines, including IL-6, IL-10, IL-21, IL-22, and IL-23. It is composed of several functional domains, with mutations in STAT3-HIES predominantly clustering in the DNA-binding domain and the Src homology 2 (SH2) domain. The SH2 domain is particularly critical for mediating protein-protein interactions, specifically in receptor binding and STAT3 dimerization—a fundamental step for its nuclear translocation and transcriptional activity. Dominant-negative mutations in this domain, such as the commonly reported p.V637M and p.R382W, result in the production of a mutant protein that competes with the wild-type protein. This competition leads to the formation of non-functional dimers, effectively reducing overall STAT3 signaling activity by 50%–90% and impairing the expression of downstream target genes (12, 13). This molecular pathogenesis underlies the key immunological defect in STAT3-HIES: a profound impairment in the differentiation of T-helper 17 (Th17) lymphocytes. The deficiency in Th17 cells and their signature cytokines, IL-17 and IL-22, explains the hallmark susceptibility to mucocutaneous candidiasis and recurrent staphylococcal infections, particularly the characteristic “cold” abscesses (14, 15). Furthermore, the dysregulation of other cytokine signals, such as the unchecked IL-4-driven IgE class switching due to impaired IL-21/STAT3 signaling, accounts for the extreme IgE elevation (5, 15). The widespread expression of STAT3 in non-immune tissues, including connective tissue, bone, and vasculature, further elucidates the multi-system nature of the syndrome, manifesting as skeletal abnormalities, retained primary dentition, and vascular anomalies (16).

The genetic variant identified in our patient, c.1915C > T (p.Pro639Ser, based on transcript NM_139276.3), is located within the SH2 domain. This specific variant was not documented in a recent and comprehensive systematic review of STAT3 defects (17), underscoring the novelty of our report and its contribution to expanding the mutational spectrum of STAT3-HIES. A particularly intriguing finding from this review was the documentation of a different nucleotide substitution at the identical codon, c.1915C > G (p.Pro639Ala), which is classified as a gain-of-function (GOF) mutation associated with a multisystem autoimmune and lymphoproliferative disease starkly contrasting with the HIES phenotype (17). This provides a crucial genetic context for our case. The p.Pro639 residue is situated within the dimerization interface of the SH2 domain. The substitution of proline with serine is predicted to introduce steric and electrostatic interference, disrupting the precise geometry required for stable homodimer formation and resulting in a dominant-negative loss of function. The fact that a proline-to-alanine change at the same residue leads to a GOF effect powerfully underscores that the functional consequence is exquisitely dependent on the specific physicochemical properties of the substituting amino acid, highlighting the structural precision of STAT3 signaling (6). The clinical presentation of our patient aligns perfectly with the established dominant-negative phenotype. Her manifestations—recurrent cold abscesses, pneumatoceles, severe eczema, and extreme IgE elevation—closely match the clinical profile of STAT3-DN patients as quantified by Hajjaligol et al., which shows a significantly higher prevalence of these features compared to STAT3-GOF patients (e.g., abscesses: 52.7% vs. 17.7%, *P* < 0.001; elevated IgE: 98.2% vs. 7.7%, *P* < 0.001) (17). The absence of the early-onset autoimmunity, lymphoproliferation, and interstitial lung disease characteristic of the STAT3-GOF syndrome further solidifies the classification of our patient's variant as dominant-negative. For a comparative overview of other pathogenic STAT3 mutations within the SH2 domain, please refer to Supplementary Table S2.

Accurate diagnosis requires a thorough differential diagnosis. The latest IUIS classification has redefined DOCK8 deficiency as a combined immunodeficiency, and other genes like ZNF341 and components of the IL-6 pathway (IL6ST, IL6R) can cause HIES-like phenotypes (7). Furthermore, conditions termed “pseudo-HIES,” such as severe atopic dermatitis, certain parasitic infections, and lymphomas, must be ruled out, as they can mimic the elevated IgE and dermatological findings (10, 18). In our patient, these alternatives were systematically excluded through detailed history, physical examination, radiographic studies (CT and MRI), histopathological analysis of abscess drainage, bone marrow examination, and targeted serological testing, thereby reinforcing the diagnosis of STAT3-HIES. The gold standard for definitive diagnosis remains next-generation sequencing to identify the causative genetic mutation.

The management of STAT3-HIES is primarily supportive and focuses on proactive infection prevention and control. Given the predisposition to staphylococcal and fungal infections, long-term antimicrobial prophylaxis is a cornerstone of therapy. Although the benefits of long-term antibiotics must be weighed against the risk of resistance, this risk is generally considered acceptable compared to the morbidity of recurrent severe infections and progressive lung damage. Our patient was managed with intravenous linezolid during acute hospitalization for a documented methicillin-sensitive Staphylococcus aureus infection and was transitioned to long-term oral trimethoprim-sulfamethoxazole prophylaxis upon discharge, which resulted in a notable reduction in the frequency of severe infections requiring hospitalization. Meticulous skin care, including the use of emollients, topical steroids, and antiseptic cleansers, is essential for managing eczema and preventing secondary infection. Our patient's skin condition improved significantly with a regimen of topical corticosteroids and diligent skin hygiene. While the total IgG level is typically normal in STAT3-HIES, some patients may benefit from immunoglobulin replacement therapy due to impaired specific antibody responses, and vaccination against Streptococcus pneumoniae is recommended (19, 20). For refractory eczema, emerging biologic therapies such as the IL-4/IL-13 receptor antagonist dupilumab have shown promising results in some patients with STAT3-HIES (21). The role of hematopoietic stem cell transplantation (HSCT) remains controversial and is typically reserved for patients with severe, progressive pulmonary disease or life-threatening complications, as it does not correct the STAT3 defect in non-hematopoietic tissues and requires careful consideration of risks and benefits (22).

### Limitations of this case report

This study has limitations. The lack of functional assays, such as analysis of Th17 cells by flow cytometry and STAT3 phosphorylation, constrained our mechanistic insight. While the genetic and clinical evidence firmly supports the diagnosis, these experiments would have directly confirmed the signaling defect. Future studies incorporating such functional analyses are warranted to fully elucidate the pathogenic impact of the STAT3 c.1915C > T variant.

## Conclusion

This case of a 6-year-old girl with a rarely STAT3 SH2 domain variant (c.1915C > T, p.Pro639Ser) underscores the critical importance of early recognition and genetic confirmation in HIES. The striking genotype-phenotype correlation observed—where this dominant-negative mutation contrasts sharply with the gain-of-function p.Pro639Ala variant at the same residue—not only expands the genetic spectrum of STAT3-HIES but also highlights the precision required for accurate diagnosis. In resource-limited settings, diagnosis may rely on characteristic clinical features and elevated IgE, yet genetic testing remains the gold standard. Long-term management focused on infection prophylaxis significantly improves patient outcomes, though targeted therapies for STAT3-HIES require further development. This case emphasizes that integrating detailed phenotyping with genetic analysis is essential for diagnosis, prognosis, and future therapeutic advances.

## Data Availability

The datasets generated and/or analyzed in this study are not publicly available due to privacy/ethical restrictions and the risk of identifying participants (case report/case series, *n* < 3). Requests for de-identified data may be directed to the corresponding author and will be considered upon reasonable request, subject to applicable institutional/ethics approvals and local data protection regulations.

## Supplementary material

Figure S1 Histopathological findings from skin abscess and bone marrow biopsy. **(A,C)** Photomicrographs of tissue from the right upper arm abscess (H&E staining). **(A)** At 200× magnification, the section shows hyperplastic fibrous and adipose tissue with granulation tissue formation, dense mixed inflammatory cell infiltration, microabscess formation (arrow), and scattered eosinophils. **(C)** At 400× magnification, a higher-power view highlights the inflammatory infiltrate, including histiocytic proliferation (arrowhead) and numerous eosinophils. **(B,D)** Photomicrographs of bone marrow trephine biopsy (H&E staining). **(B)** At 200× magnification, the marrow is hypercellular with a marked increase in eosinophil precursors. **(D)** At 400× magnification, a higher-power view confirms a prominent eosinophilic hyperplasia.
