# Supplementary figures and images for: STAT3 c.1915C > T variant-associated Hyper-IgE syndrome in a child: a case report
Source: Front Pediatr. 2025 Dec 12;13:1693297. doi: 10.3389/fped.2025.1693297 (PMC12741137; doi:10.3389/fped.2025.1693297)

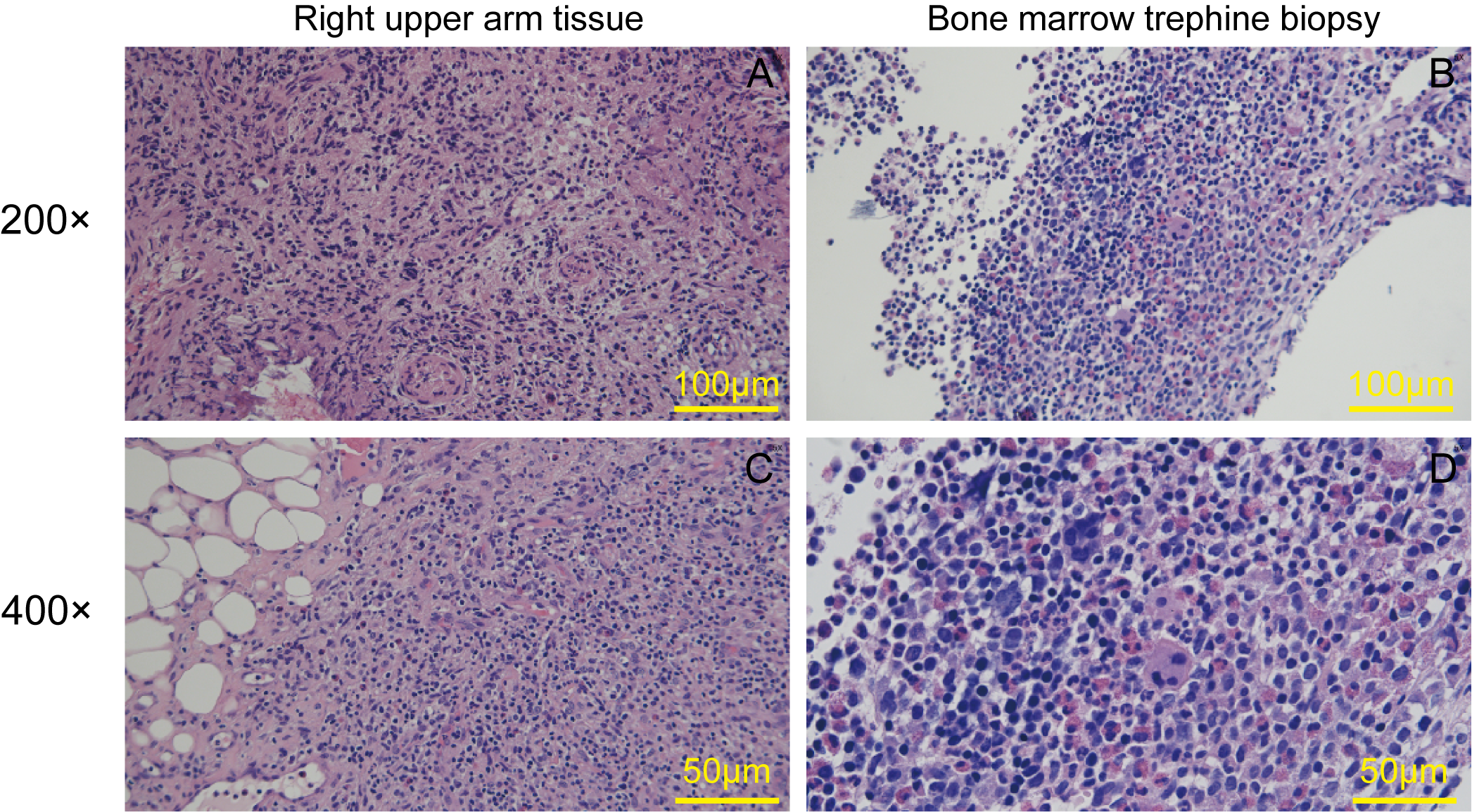

Supplement: Figure S1 — Histopathological findings from skin abscess and bone marrow biopsy. (A,C) Photomicrographs of tissue from the right upper arm abscess (H&E staining). (A) At 200× magnification, the section shows hyperplastic fibrous and adipose tissue with granulation tissue formation, dense mixed inflammatory cell infiltration, microabscess formation (arrow), and scattered eosinophils. (C) At 400× magnification, a higher-power view highlights the inflammatory infiltrate, including histiocytic proliferation (arrowhead) and numerous eosinophils. (B,D) Photomicrographs of bone marrow trephine biopsy (H&E staining). (B) At 200× magnification, the marrow is hypercellular with a marked increase in eosinophil precursors. (D) At 400× magnification, a higher-power view confirms a prominent eosinophilic hyperplasia. [file Image1.tif]
