# Supplementary material for: STAT3 c.1915C > T variant-associated Hyper-IgE syndrome in a child: a case report
Source: Front Pediatr. 2025 Dec 12;13:1693297. doi: 10.3389/fped.2025.1693297 (PMC12741137; doi:10.3389/fped.2025.1693297)
Supplement: Supplementary file 2 [file Table1.docx]

**Table S1 Key Laboratory Findings**

| **Test** | **Result** | **Interpretation** | **Reference range** | **Units** |
| --- | --- | --- | --- | --- |
| RBC count | 4.06 | Low | 4.2~5.7 | 10^12^/L |
| WBC count | 23.59 | High | 4.3~11.3 | 10^9^/L |
| Neutrophils count | 9.03 | High | 1.6~7.8 | 10^9^/L |
| Lymphocytes count | 8.54 | High | 1.5~4.6 | 10^9^/L |
| Monocytes count | 1.20 | High | 0.13~0.76 | 10^9^/L |
| Eosinophils count | 4.47 | High | 0~0.68 | 10^9^/L |
| Basophils count | 0.07 | Normal | 0~0.07 | 10^9^/L |
| Haemoglobin | 97 | Low | 118~156 | g/L |
| PLT | 401 | Normal | 167~453 | 10^9^/L |
| Immunoglobulin E | 22800 | High | 0~165 | IU/mL |
| CRP | 15.9 | High | 0~10 | mg/L |
| IL-6 | 45.3 | High | ＜7 | pg/mL |
| PCT | 0.07 | High | 0~0.05 | ng/mL |
| CD3⁺ T cells | 75 | Normal | 58~86 | % |
| CD3⁺/CD4⁺ T cells | 49.3 | Normal | 27~51 | % |
| CD3⁺/CD8⁺ T cells | 21.1 | Normal | 19.7~38.9 | % |
| CD4+/CD8+ ratio | 2.34 | Normal | 0.71~2.78 | Not applicable |
| Total B lymphocytes | 22.3 | High | 5~20 | % |
| NK cells | 2.1 | Low | 3.3~22.5 | % |
| Parasite 7-IgG panel | Negative | Negative | Negative | Not applicable |
| Chest CT | A pneumatocystic lesion in the right upper lobe | Positive | Negative | Not applicable |
| MRI of soft tissue |  | Positive | Negative | Not applicable |
| Bacterial culture (pus) | Staphylococcus aureus (MRSA) | Positive | Negative | Not applicable |
| ltrasound of superficial lymph nodes throughout the body | Bilateral cervical and axillary lymphadenopathy, likely reactive | Positive | Negative | Not applicable |
| Pathology report (R upper-arm mass) | Fibrofatty proliferation with granulation tissue, mixed acute/chronic inflammation, microabscesses, scattered eosinophils, and histiocytic hyperplasia | Positive | Negative | Not applicable |
| Bone marrow trephine biopsy | Eosinophils increased significantly | Positive | Negative | Not applicable |
