# Supplementary material for: STAT3 c.1915C > T variant-associated Hyper-IgE syndrome in a child: a case report
Source: Front Pediatr. 2025 Dec 12;13:1693297. doi: 10.3389/fped.2025.1693297 (PMC12741137; doi:10.3389/fped.2025.1693297)
Supplement: Supplementary file 3 [file Table2.docx]

**Table S2: Key Pathogenic STAT3 Mutations in the SH2 Domain and Associated Phenotypes**

| **Nucleotide Change** | **Protein Change** | **Functional Effect** | **Key Clinical Features** |
| --- | --- | --- | --- |
| c.1771A>G | K591E | Presumed dominant-negative (DN), impairing STAT3 dimerization and signaling. | Classic HIES phenotype: recurrent pneumonia, newborn rash, pathologic fractures, characteristic facies, high palate, very high IgE. |
| c.1865C>T | T622I | Presumed dominant-negative (DN), impairing STAT3 function. | Classic HIES phenotype, may include lung cysts, eczema, candidiasis. |
| c.1907C>A | S636Y | Presumed dominant-negative (DN), impairing STAT3 function. | Classic HIES phenotype, recurrent skin abscesses, lung cysts, eosinophilia. |
| c.1909G>A | V637M | Common mutation;  dominant-negative (DN), impairing STAT3 phosphorylation and nuclear translocation. | Classic HIES phenotype, recurrent pneumonia, skin infections, characteristic facies, high palate. |
| c.1910T>C | V637A | Presumed dominant-negative (DN), impairing STAT3 function. | Classic HIES phenotype, recurrent infections, eczema, lung cysts. |
| c.1915T>C | P639S | Presumed dominant-negative (DN), impairing STAT3 function. | Classic HIES phenotype, recurrent pneumonia, newborn rash, pathologic fractures. |
| c.1915C>G | p.P639A | Presumed  Gain-of-Function (GOF) | STAT3-GOF Syndrome: Early-onset multi-organ autoimmunity (55.6%), lymphoproliferation (47.7%), interstitial lung disease (9.8%). Not typically associated with extremely high IgE (7.7%) or pneumatoceles (4.6%). |
| c.1939A>G | p.N647D | Presumed  dominant-negative (DN) | Classic STAT3-DN/HIES: Recurrent sinopulmonary infections, eczema, and elevated IgE. |
| c.1954G>A | p.E652K | Presumed  dominant-negative (DN) | Classic STAT3-DN/HIES: Recurrent infections, dermatological manifestations, and elevated IgE. |
| c.1970A>G | Y657C | Presumed dominant-negative (DN), impairing STAT3 function. | Classic HIES phenotype, recurrent skin abscesses, lung cysts, eosinophilia. |
| c.2003C>T | S668F | Presumed dominant-negative (DN), impairing STAT3 function. | Classic HIES phenotype, recurrent pneumonia, characteristic facies, very high IgE. |
| c.1915T>C^*^ | P639S | Presumed dominant-negative (DN), impairing STAT3 function. | Classic HIES phenotype, (This Study): Recurrent "cold" skin abscesses, pneumatoceles, severe eczema, extremely elevated IgE (22,800 IU/mL), characteristic facies, and growth delay. Absence of predominant autoimmunity. |

The asterisk (**^*^**) indicates the variant and clinical features described in this study.
